# BD SurePath Direct to Slide (DTS) cervical cytology: Migrating the benefits of liquid-based cytology to low-resource settings

**DOI:** 10.1093/ajcp/aqae068

**Published:** 2024-06-24

**Authors:** Douglas P Malinowski, Ryan Callaghan, Clark Whitehead, Romana Nelson, Lisa Allen, Dondrea Purnell, Adriann Taylor, Didier Morel, Aisha Dhewar, Vanessa Soh Chai-Hong, Goh Giap-Hean, Soo-Yong Tan, Sagar Tapas, Jeffrey Andrews

**Affiliations:** Becton, Dickinson and Company, BD Life Sciences–Integrated Diagnostic Solutions, Sparks, MD, US; Becton, Dickinson and Company, BD Life Sciences–Integrated Diagnostic Solutions, Sparks, MD, US; Becton, Dickinson and Company, BD Life Sciences–Integrated Diagnostic Solutions, Sparks, MD, US; Becton, Dickinson and Company, BD Life Sciences–Integrated Diagnostic Solutions, Sparks, MD, US; Becton, Dickinson and Company, BD Life Sciences–Integrated Diagnostic Solutions, Sparks, MD, US; Becton, Dickinson and Company, BD Life Sciences–Integrated Diagnostic Solutions, Sparks, MD, US; Becton, Dickinson and Company, BD Life Sciences–Integrated Diagnostic Solutions, Sparks, MD, US; Becton, Dickinson and Company, BD Life Sciences–Integrated Diagnostic Solutions, Sparks, MD, US; Becton, Dickinson and Company, BD Life Sciences–Integrated Diagnostic Solutions, Sparks, MD, US; National University Hospital, Singapore; National University Hospital, Singapore; National University Hospital, Singapore; Lifeline Labs, New Delhi, India; Becton, Dickinson and Company, BD Life Sciences–Integrated Diagnostic Solutions, Sparks, MD, US

**Keywords:** SurePath, cervical cytology, early detection of cancer, uterine cervical neoplasms, equipment and supplies, reproducibility of results, cytological techniques

## Abstract

**Objectives:**

The benefits of liquid-based cytology (LBC) in routine cervical cancer screening are often associated with the availability of instrumented platforms and economic considerations. A low-cost alternative to LBC in low-volume settings remains an unmet need.

**Methods:**

A multisite evaluation of the BD SurePath (SurePath) LBC Direct to Slide (DTS) method was conducted. The DTS preparations were evaluated across 3 sites. Cytology features for DTS preparation included predetermined thresholds for total cellularity, cell distribution, cellular preservation, and stain quality. Rare event detection was evaluated using SiHa cells spiked into pools from negative cytology specimens. Concordance between Bethesda classification results was evaluated for SurePath LBC and DTS methods using routinely collected SurePath specimens in a split-sample study design.

**Results:**

The DTS specimens met criteria for total cellularity, cell distribution, cellular preservation, and stain quality in more than 98% of all cases. Rare event detection was observed with an average detection of 5 SiHa cells per 2 mL of specimen. Concordant cervical cytology classifications were observed between SurePath LBC and DTS methods.

**Conclusions:**

The results demonstrate that the DTS process is suitable for routine cervical cytology evaluation. The procedure is reproducible and detected abnormal cervical cells in concordance with standard SurePath LBC preparation.

**Key Points**

- The Direct to Slide (DTS) preparation method produced cervical cytology slides using SurePath liquid-based cytology (LBC) specimens that are suitable for routine cervical cytology evaluation.
- The DTS preparation met acceptance criteria for cellularity, cell distribution, cellular preservation, and stain quality. The overall concordance of acceptable results (across 3 sites) was more than 98%.
- DTS detected abnormal cervical cytology using routinely collected SurePath LBC specimens; a split-sample study demonstrated that DTS and SurePath processing provided similar diagnoses.

## INTRODUCTION

Cervical cancer is the fourth most common cancer in women by incidence and mortality, worldwide, with an estimated 342,000 deaths and 604,000 new cases in 2020.^1^ Although its incidence and associated mortality is declining in countries that have adopted an organized cervical cytology screening program, it remains the most commonly diagnosed cancer in 28 countries and the leading cause of cancer death in 42 countries.^2,3^

Cervical cancer is a slowly progressing disease with well-defined precursor lesions (dysplasia or squamous intraepithelial lesions), which can be detected years before cancer develops.^4^ Based on these features, screening has proven to be highly effective in the detection of cervical cancer and its malignant precursors during routine clinician visits. When abnormalities are detected, subsequent patient referral to colposcopy and appropriate treatment have resulted in reductions in both incidence and mortality.^5,6^ Historically, cervical cancer screening relied on cervical cytology, where exfoliated cells, collected from the uterine cervix, were examined under the microscope for the presence of abnormal cells.^7^ It is now recognized that the human papillomavirus (HPV) is the etiological agent responsible for most cervical cancer cases.^4^ This knowledge has led to a change in the approach toward cervical cancer screening, in which molecular-based testing is used to detect high-risk HPV infection (which carries an increased risk for developing cervical precancer), as well as cervical cytology to help ascertain the degree of cellular abnormality associated with HPV infections.^8,9^ Both determinations are now included in cervical cancer screening guidelines and subsequent clinical management recommendations.^10,11^

During the past 2 decades, the use of liquid-based cytology (LBC) has replaced the conventional Papanicolaou (Pap) smear in many countries around the world.^12^ The advantage of LBC technology is that it not only affords the use of cytology to detect cellular abnormalities but also serves as a medium for cellular preservation and the use of HPV testing, thereby permitting a single specimen collection to be used for both HPV testing as well as cytology.^13^ There are 2 commercially available LBC systems approved for use in cervical cancer screening by the US Food and Drug Administration (FDA): the ThinPrep Pap Test with PreservCyt Solution (Hologic) and the BD SurePath Liquid-Based Pap Test (SurePath LBC; Becton, Dickinson and Company).^14,15^ Both of these LBC systems are FDA approved for use with 1 or more high-risk HPV assays, permitting an integrated solution for both HPV and cytology testing in routine cervical cancer screening programs.

The advantages of LBC systems have been reviewed elsewhere,^16^ and these systems are routinely used in many laboratories in North America and globally. The benefits of LBC include

- Standardized collection and processing
- Unique predefined regions on the slide to screen
- Well-preserved morphology
- Specimens compatible with imaging-assisted screening
- Compatible with molecular tests such as high-risk HPV assays

One of the characteristics of LBC systems is the reliance on an automated system (eg, BD Totalys SlidePrep; Becton Dickinson and Company) to process the LBC specimen for slide preparation, staining, and subsequent examination.^17^ The economics of LBC adoption within a laboratory depend on the annual volume of cervical cytology specimens processed in the laboratory. Thus, LBC systems are cost-effective in large-volume laboratories but are less likely to be cost-effective or affordable in low-volume settings.^18,19^ In many countries around the world, this volume dependency makes LBC adoption cost difficult to justify and implement. Without the ability to take advantage of LBC technology, these laboratories continue to rely on the conventional Pap smear as the method for cytology examination. This further separates HPV testing and cervical cytology examination and requires 2 separate specimens from each patient.

To address this issue, we developed a manual LBC preparation method predicated upon the SurePath method (BD SurePath Direct to Slide, or DTS; Becton, Dickinson and Company). The DTS process incorporates the cell enrichment advantages of SurePath LBC, using only a modified slide holder and common tabletop cytology centrifuge. This simplified method lowers the cost to produce a high-quality LBC slide while retaining the advantages of the SurePath LBC preservative.^20^

In this study, we report on slide quality features of the DTS processing method, the reproducibility of cervical slide preparation using the DTS process, rare event detection, and preliminary demonstration of equivalency between DTS slides and standard SurePath LBC slides.

## METHODS

SurePath LBC specimens were prospectively collected from patients attending a routine cervical cancer screening clinic. The use of these cervical cytology specimens was approved by local institutional review boards, and written consent was obtained prior to any study-related activity. SiHa cell lines were obtained from ATCC and propagated using standard cell culture techniques.

### DTS Process

The DTS method was developed to require only a standard benchtop centrifuge and is able to produce about 6 slides in 10 minutes. DTS slide holders were designed to fit standard centrifuge bucket dimensions. A single slide holder fits a standard SurePath LBC centrifuge bucket, and the triple slide holder fits a 96-well microtiter plate centrifuge bucket.

The BD SurePath Direct to Slide kit was used in this study. Throughout the article, we have used the term Direct to Slide (or DTS) to refer to this processing methodology. The DTS slide preparation was performed according to the manufacturer’s recommended procedure. Briefly, slides were produced by using modified holders that secure a BD PreCoat slide (Becton Dickinson and Company) and a standard BD Settling Chamber (“settling chamber”; Becton Dickinson and Company). Then, 1 mL BD Density Reagent (“density reagent”; Becton Dickinson and Company) was placed onto each settling chamber, and 2 mL SurePath LBC specimen was then layered on top of the density reagent. The specimens were centrifuged for 2 minutes at 200*g*, decanted, washed with alcohol, and manually stained using standard Pap staining methodology. In the rare event portion of the study, this same method was used to prepare 48 slides from a pool that was constructed by spiking cultured SiHa cells into a pool of normal (negative for intraepithelial lesions or malignancies [NILM]) cervical samples at a concentration of 1:6025.

### DTS Slide Feature Assessment

The DTS slides were prepared using SurePath LBC preservative fluid from women attending a routine cervical cancer screening program. Comparisons of cytology features using the DTS method were made between 3 sites. Site 1 performed an initial feasibility study (n = 96 cases) and then a second validation study (n = 384 cases). Site 2 conducted a similar study using a prospectively collected set of SurePath LBC specimens (n = 300 cases). At both sites, the following slide features were assessed: total cellularity, cell distribution, cellular preservation, and stain quality (using predetermined criteria for slide acceptance) TABLE 1. Each DTS slide was assessed for these features. A score of 1 or 2 was considered acceptable for cellular preservation, cell distribution, and stain quality, while a score of 1 to 3 was considered acceptable for total slide cellularity. Abnormal cervical cytology results were noted during evaluation of cytology features from routinely collected SurePath LBC specimens processed using the DTS preparation method.

**TABLE 1:** Cellular Features and Acceptance Criteria for Direct to Slide Prepared Slides

Cellular feature	Grading score	Description	Acceptable (Yes/No)
Cellularity	1	>90,000 cells (181+ cells per high-powered field)	Yes
	2	>40,000-90,000 cells (81-180 cells per high-powered field)	Yes
	3	5000-40,000 cells (10-80 cells per high-powered field)	Yes
	4	<5000 (8-10 cells per high-powered field)	No
Cell distribution	1	>75% even cell distribution (0%-25% of slide shows uneven distribution)	Yes
	2	>50% but <75% even cell distribution (25%-50% of slide shows uneven distribution)	Yes
	3	<50% even cell distribution (50%-100% of slide shows uneven distribution)	No
Cellular preservation	1	>90% of cells display sharp nuclear and cytoplasmic features in single cells and clusters (0%-10% of cells show poor preservation)	Yes
	2	75%-90% of cells display sharp nuclear and cytoplasmic features in single cells and clusters (10%-25% of cells show poor preservation)	Yes
	3	<75% of cells display sharp nuclear and cytoplasmic features in single cells and clusters (25%-100% of cells show poor preservation)	No
Stain quality	1	>90% stain uniformity or stain intensity (slightly light or dark areas) or stain quality (good nuclear/cytoplasmic contrast) observed (0%-10% of slide area affected by any or all of these features)	Yes
	2	75%-90% stain uniformity, stain intensity, and/or stain quality observed (10%-25% of slide area affected by any or all of these features)	Yes
	3	<75% stain uniformity, stain intensity, or stain quality observed (25%-100% of slide area affected by any or all of these features)	No

### Preparation of DTS and Traditional SurePath LBC Slides From Clinical Specimens—Split-Sample Study

Site 3 conducted a similar study using prospectively collected cervical cytology specimens from 500 patients who were presented for routine cervical cytology screening. Sample collection from the cervix was performed using a Rovers Cervex Brush (“Cervex Brush”; Rovers Medical Devices), and the entire brush head was washed in SurePath LBC preservative to create a cell suspension, as described by the manufacturer.^14^ Then, 8 mL of the collected SurePath LBC specimen was used for processing and staining conducted as per routine laboratory protocol. The remaining 2 mL of sample in the BD SurePath preservative vial was processed using the modified DTS protocol.

Both SurePath LBC and DTS slides were examined by a qualified cytotechnologist (V.S.C.). Where slide discordance was noted, the case was reevaluated by a cytopathologist (G.G.H.) for final diagnostic assessment. Cytology slides in both groups were assessed in a blinded fashion for cellularity, cell distribution, cellular preservation, and stain quality, based on a similar set of criteria as detailed for sites 1 and 2 TABLE 1. Both slide types between the split SurePath and DTS prepared slides were evaluated for cervical morphology and abnormal specimens (n = 19 cases identified from 500 collected specimens) using the Bethesda classification.^21^ The DTS and BD SurePath LBC specimens were independently evaluated for Bethesda classification with the results from the SurePath LBC evaluation blinded during the DTS preparation evaluation.

### Rare Event Detection Using SurePath DTS

A pool of NILM cervical specimens in SurePath was created by combining residual SurePath LBC specimens following routine assessment from cervical cancer screening programs. The NILM pool contained on average approximately 500,000 cells/10 mL of SurePath preservative fluid. This NILM pool was spiked with cultured SiHa cells to create a concentration of approximately 100 SiHa cells/10 mL of cervical NILM pool. This SiHa pool was processed using the DTS method, and the average number of SiHa cells per slide was recorded.

### Statistical Analysis

Two-sided 95% confidence intervals were computed using Wilson’s score method as implemented in function binconf of package Hmisc with R version 3.3.2. Additional confidence intervals were calculated using the Microsoft Excel statistics analysis package. Proportions agreement was calculated using MedCalc online software.

## RESULTS

### Cytology Acceptance Criteria Following DTS and Site-to-Site Reproducibility

**FIGURE 1:**
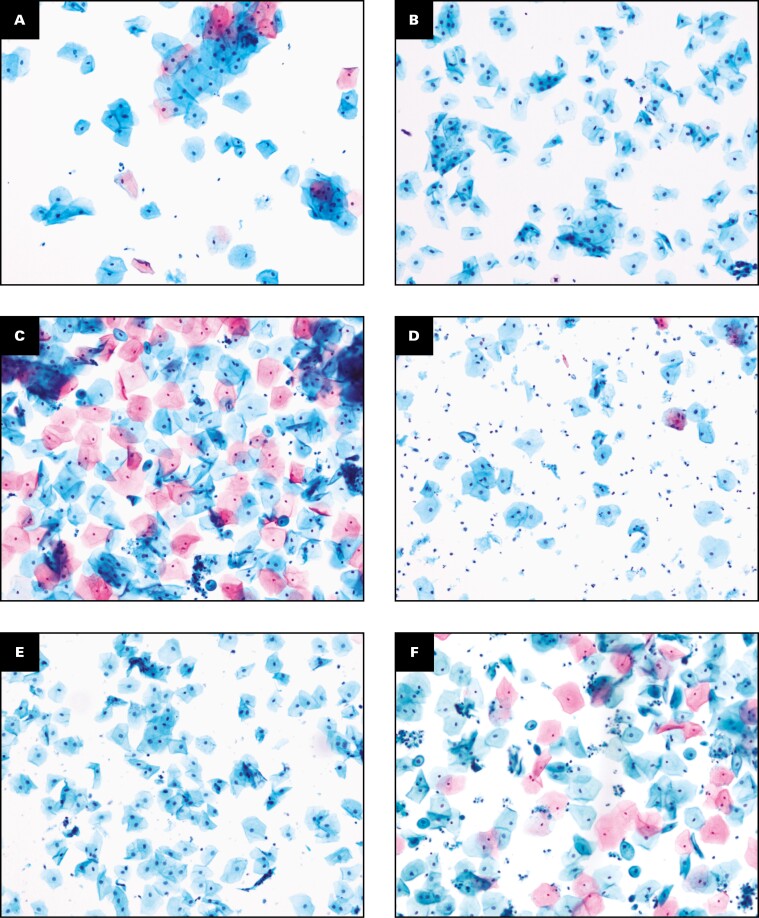
Comparison of cytology features between Direct to Slide (DTS) (**A-C**) and SurePath liquid-based cytology (LBC) (**D-F**) preparation in the split-sample study (×10). SurePath LBC preparation methodology. The remaining residual specimen volume in the vial was reprocessed using the DTS preparation methodology. Both slides were examined. Representative results for specimens showing inflammatory cells, blood, the human papillomavirus effect (koilocytosis), high-grade squamous intraepithelial lesion, and adenocarcinoma cells are shown. The effect of blood and inflammatory cells appeared more pronounced in DTS preparations than the SurePath LBC methodology. Both DTS and SurePath displayed equivalent morphology presentation of abnormal cervical cells.

Using the preparation method as described in the manufacturer’s instructions for use, DTS slides appear similar to standard SurePath LBC slides prepared using instrumented systems FIGURE 1. Through inspection, the DTS slides are similar in terms of cellularity, cell distribution, cellular preservation, and stain quality when compared with the same specimens processed using standard, instrumented, SurePath LBC preparation. A feasibility study (n = 96) was conducted at site 1 to evaluate these cytology features (Supplementary Table 1; all supplementary material is available at *American Journal of Clinical Pathology* online). As shown, DTS met acceptance criteria TABLE 1 for cellularity, cellular preservation, and stain quality for 100% of the slides and for 99% of the slides regarding the cell distribution criterion.

The feasibility study at site 1 was followed by larger studies at sites 1, 2, and 3. The acceptance rate for DTS slides across the 4 criteria is shown in TABLE 2. As shown, the percentages of slides with acceptable scores for cellularity, cell distribution, and cellular preservation were all high and ranged from 98.4% to 99.7%, 99.4% to 100%, and 99.7% to 100%, respectively. The slight differences observed between the sites in terms of cellularity and cell distribution acceptance rates were not statistically significant (all *P* values were >.05). All 3 sites recorded 100% acceptance for stain quality. Variability between sites was low (no greater than 1.3%) for all 4 criteria. However, given the higher variation observed in the cellularity acceptance rates compared with the other 3 criteria, further analysis was performed for sites 1 and 2 to examine results by the 4 ranges used to determine cellularity acceptance (Supplementary Table 2). The additional analysis on cellularity showed that 94% to 99.9% of slides displayed more than 5,000 to 90,000 cells, and 21.6% to 77.0% of slides displayed more than 40,000 to more than 90,000 cells.

**TABLE 2:** Reproducibility of Direct to Slide Preparation and Acceptance Rates for Slide Quality Features in Multicenter Evaluation

	Acceptance rate, % (No.)						
Slide quality features	Site 1 (n = 384)	Site 2 (n = 300)	Site 3 (n = 500)	Interlaboratory comparison	Difference, %	95% CI	*P* value
Cellularity	99.7 (383)	99.3 (298)	98.4 (492)	Site 1 vs site 2	0.4	–0.9251 to 2.1657	.4508
				Site 1 vs site 3	1.3	–0.1570 to 2.8441	.0585
				Site 2 vs site 3	0.9	–1.0180 to 2.5057	.2700
Cell distribution	100 (384)	100 (300)	99.4 (497)	Site 1 vs site 2	0	–1.2643 to 0.9905	NA
				Site 1 vs site 3	0.6	–0.4666 to 1.7490	.1286
				Site 2 vs site 3	0.6	–0.7248 to 1.7490	.1792
Cellular preservation	100 (384)	99.7 (299)	100 (500)	Site 1 vs site 2	0.3	–0.7217 to 1.8075	.2832
				Site 1 vs site 3	0	–0.7624 to 0.9905	NA
				Site 2 vs site 3	0.3	–0.5026 to 1.8075	.2207
Stain quality	100 (384)	100 (300)	100 (500)	Site 1 vs site 2	0	–1.2643 to 0.9905	NA
				Site 1 vs site 3	0	–0.7624 to 0.9905	NA
				Site 2 vs site 3	0	–0.7624 to 1.2643	NA

NA, not applicable.

### Split SurePath LBC Sample Comparison With DTS

Site 3 conducted a split-sample (n = 500 each for DTS and SurePath LBC) study, which allowed a relative comparison between DTS and SurePath LBC regarding acceptance rate for the 4 cytology criteria. Results from this comparison showed no difference between DTS and SurePath LBC for cell distribution, cellular preservation, and stain quality. There was a 1.4% difference between DTS and SurePath LBC (98.4% vs 99.8%, respectively) for cellularity TABLE 3. For the cellularity analysis in the split-sample study, the 8 discordant DTS specimens were associated with scant cellularity and accompanying inflammation and obscuring white blood cells. One of these discordant DTS cases with scant cellularity was also observed in the discordant SurePath LBC specimen TABLE 3 (Supplementary Figure 1). For the cell distribution analysis in the split-sample study, there were 3 unacceptable DTS specimens associated with obscuring blood, obscuring inflammatory cells, or thick cellular overlap TABLE 3 (Supplementary Figure 2). Although these differences were noted, the acceptance criteria of more than 95% agreement between SurePath LBC and DTS preparations were met. Overall, the concordance of DTS and SurePath LBC preparations during the evaluation of total cellularity, cell distribution, cellular preservation, and stain quality was more than 98.6% (Supplementary Table 3).

**TABLE 3:** Split-Sample Comparison of Slide Quality Acceptance Rates Between SurePath and DTS

Characteristic	DTS slides (n = 500), % (No.)	SurePath slides (n = 500), % (No.)	Difference, %	95% CI	*P* value
Cellularity	98.4 (492)^a^	99.8 (499)^b^	1.4	0.1862 to 2.9340	.0191
Cell distribution	99.4 (497)^c^	100 (500)	0.6	–0.2590 to 1.7490	.0830
Preservation	100 (500)	100 (500)	0	–0.7624 to 0.7624	NA
Stain quality	100 (500)	100 (500)	0	–0.7624 to 0.7624	NA

DTS, Direct to Slide; NA, not applicable.

^a^Eight unacceptable DTS samples included 6 cases involving scant cells (5 also with inflammatory/obscuring inflammatory cells), 1 case involving obscuring inflammatory cells, and 1 case involving obscuring inflammatory cells with some red blood cells.

^b^One unacceptable SurePath liquid-based cytology sample showed scant cellularity.

^c^Three unacceptable DTS samples were associated with obscuring blood, obscuring inflammatory cells, or thick cellular overlap.

### Detection of Abnormal Cases Using the DTS Preparation Method During the Validation and Split-Sample Studies

**FIGURE 2:**
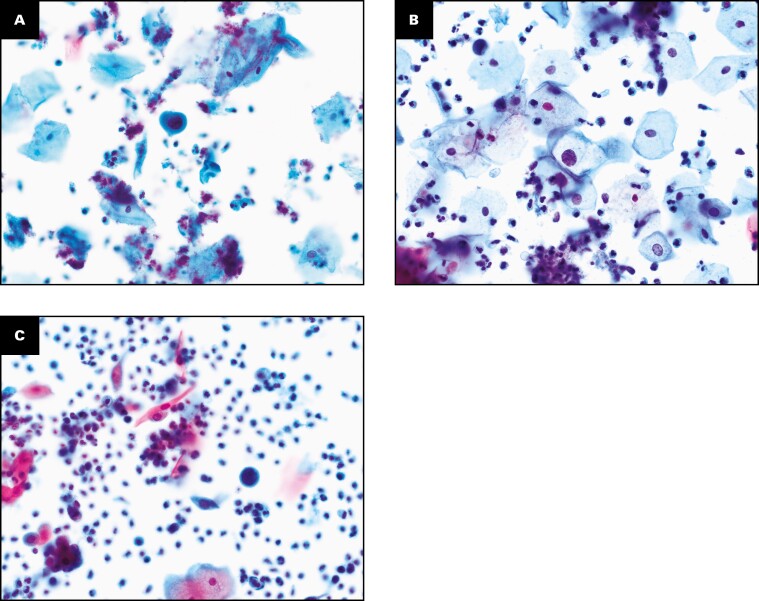
Detection of abnormal cases using the Direct to Slide (DTS) preparation method during the validation study (×20). Abnormal cervical cytology classification was noted using Bethesda classification categories for the DTS and SurePath liquid-based cytology methods. During this validation, cervical abnormalities were observed, including atypical squamous cells—cannot rule out high-grade lesion (**A**), low-grade squamous intraepithelial lesion (**B**), and cervical squamous cell carcinoma (**C**).

During the DTS validation study at site 1 (n = 394 SurePath specimens), cases of abnormal cytology cells were identified. The cervical cytology classification results from these studies were noted using Bethesda classification categories, although no direct comparison was made between the DTS preparation method and a separate SurePath LBC slide from the same specimen. During this validation, cervical abnormalities were observed, including low-grade squamous intraepithelial lesion (LSIL), atypical squamous cells—cannot rule out high-grade lesion (ASC-H), and 1 case of cervical squamous cell carcinoma FIGURE 2.

**FIGURE 3:**
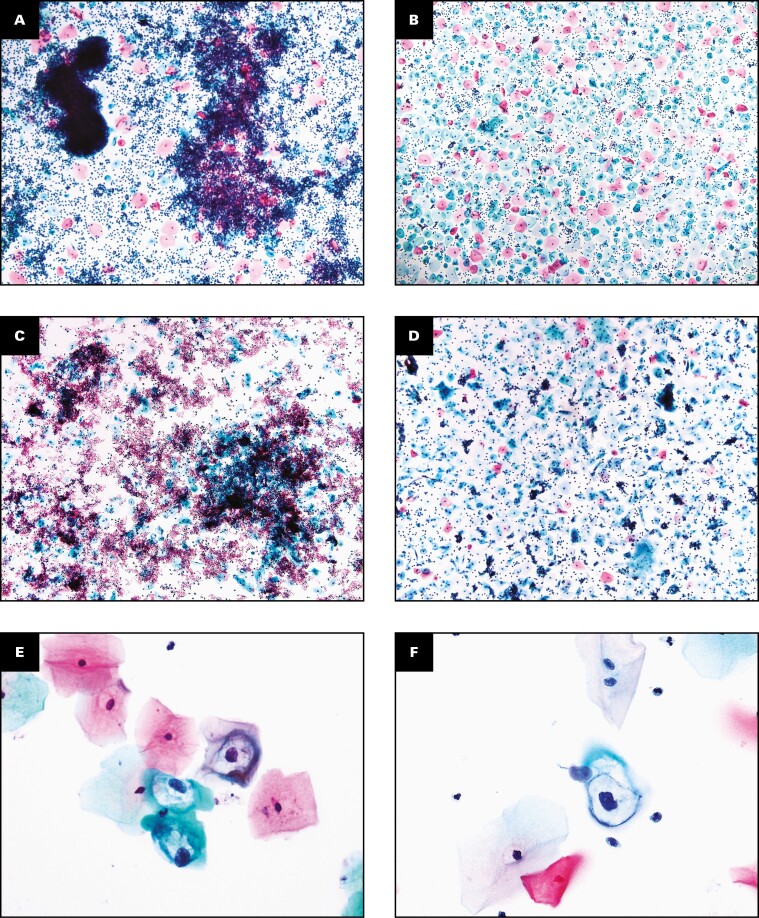

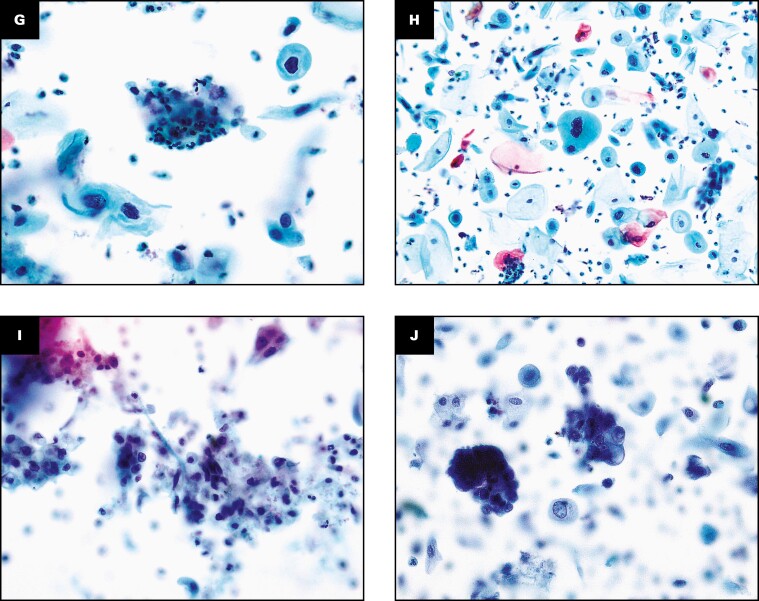
Detection of abnormal cases using the Direct to Slide (DTS) preparation method during the split-sample study. Abnormal cervical cytology classification was noted using Bethesda classification categories for the DTS (**A**, **C**, **E**, **G**, **I**) and SurePath liquid-based cytology (**B**, **D**, **F**, **H**, **J**) methods. Both preparation types showed representative results for specimens showing inflammatory cells (**A**, **B**), blood (**C**, **D**), koilocytosis (**E**, **F**), high-grade squamous intraepithelial lesion (**G**, **H**), and adenocarcinoma cells (**I**, **J**). The images of blood and inflammatory cases are shown at ×4 magnification. The images of koilocytes are shown at ×40 magnification. The images of high-grade squamous intraepithelial lesion and adenocarcinoma cases are shown at ×20 magnification.

The cervical cytology classification results from the split-sample DTS and SurePath LBC preparations were compared using Bethesda classification categories. Both preparation types were examined, and representative results for specimens showing inflammatory cells, blood, the HPV effect (koilocytosis), high-grade squamous intraepithelial lesion (HSIL), and adenocarcinoma cells are shown in FIGURE 3. The effect of blood and inflammatory cells appeared more pronounced in DTS preparations than SurePath LBC slides. Both DTS and SurePath displayed an equivalent morphologic presentation of abnormal cervical cells. Overall, there was good agreement between SurePath LBC and DTS preparation methods in terms of the reported cytology results distribution TABLE 4. There were 19 abnormal cytology results (including cases of LSIL, ASC-H, and cervical adenocarcinoma) in the split-sample study TABLE 4 and FIGURE 3. All abnormal cases detected relied on the final cytology evaluation as the final result. No attempt was made to confirm cytology-based diagnosis with histopathology-confirmed diagnosis using cervical biopsy specimens.

**TABLE 4:** Comparison of Bethesda Cytology Classification Results in DTS-SurePath Split-Sample Study

DTS preparation	SurePath LBC preparation, No.						
	Unsat	NILM	Low grade^a^	High grade^b^	Adenocarcinoma	AGUS	Total
Unsat	1	7					8
NILM		473					473
Low grade^a^			10				10
High grade^b^				6			6
Adenocarcinoma						1	1
AGUS					2		2
Total	1	480	10	6	2	1	500

AGUS, atypical glandular cells of undetermined significance; ASC-H, atypical squamous cells—cannot rule out high-grade lesion; ASCUS, atypical squamous cells of undetermined significance; DTS, direct to slide; HSIL, high-grade squamous intraepithelial lesion; LBC, liquid-based cytology; LSIL, low-grade squamous intraepithelial lesion; NILM, negative for intraepithelial lesion or malignancy; Unsat, unsatisfactory.

^a^ASCUS/LSIL.

^b^ASC-H/HSIL.

### Rare Event Detection Using SiHa Cell Recovery

To better evaluate the DTS preparation method for efficient capture and detection of rare cell events, such as might be encountered in routine evaluation of cervical cytology specimens, we conducted an experiment using spiked SiHa cells (ratio of SiHa to SurePath LBC media = 1:6250) in normal or NILM SurePath LBC specimens as a surrogate for rare cell detection. These spiked specimens were processed using the standard DTS preparation method. We expected to observe 1 to 2 SiHa cells per DTS preparation. The observed detection rates of SiHa cells in the DTS preparations ranged from 2 to 12 SiHa cells per DTS preparation, with an average detection of 5 SiHa cells per DTS slide (Supplementary Figure 3). This observation indicates that the DTS preparation method has the ability to detect rare event cells contained within SurePath LBC specimens.

### Detection of Microorganisms in SurePath LBC Specimens Using the DTS Method

During the split-sample study at site 3, infectious microorganisms were detected in both the SurePath LBC and DTS slides, including 13 cases of *Candida*, 1 case of *Actinomyces*, and 1 case of *Trichomonas vaginalis* (Supplementary Table 4 and Supplementary Figure 4). Although the validation and split-sample studies were not designed to compare the accuracy and concordance of infectious organism detection using the DTS method, these data suggest that the DTS preparation method is capable of detecting the presence of infectious organisms using standard morphologic assessment.

## DISCUSSION

Here, we evaluated cytology metrics following the DTS preparation method as an alternative to the traditional SurePath LBC method. We found that DTS provided a high percentage of acceptable results related to 4 cytology features: cellularity, cell distribution, cellular preservation, and stain quality. Three sites provided data regarding these 4 criteria with no significant differences (high reproducibility) in acceptance rates between the sites. Furthermore, DTS performed similarly to SurePath LBC across the 4 criteria, with the only differences occurring in cellularity (1.4% difference) and cell distribution (0.6% difference). Furthermore, cytology scoring by the Bethesda system in DTS and SurePath LBC slides showed results concordance between the methods. By visual inspection, DTS was similar to SurePath LBC, and abnormal cytology samples were identified from split SurePath specimens with both preparation methods. Using contrived specimens spiked with SiHa cells, DTS was able to detect rare events, which implies the feasibility of identifying high-grade lesions and cancer using the DTS preparation method. Finally, the DTS preparation method facilitates identification of infectious microbes (eg, *Candida* spp and *Trichomonas vaginalis*), which may benefit the health of underserved women in areas beyond cervical cancer screening.

Screening is widely recognized as an effective approach for reducing the incidence and mortality of cervical cancer. The success of cervical cancer screening was historically based on the use of the conventional Pap smear. Newer preparation technologies such as LBC offer equivalent or better clinical performance for the detection of cytologic abnormalities and a lower rate of unsatisfactory specimens.^16^ Moreover, the inclusion of HPV testing further increases the clinical sensitivity to detect cervical precursor lesions in the screening population. Although HPV- and LBC-based technologies for cervical cancer screening and triage methods are now recognized as recommended approaches for routine screening,^10,11^ successful implementation requires a minimum volume of annual cervical cancer screening to make the technology affordable and cost-effective in the laboratory. For laboratories and clinics with a low annual volume of cervical cancer screening, these more advanced technologies are not easily implemented or cost justified. Thus, the cost of LBC instrumentation can be a barrier that deprives both the laboratory and the patients served of access to these improvements in cervical cancer screening. Importantly, the acceptance rates for the 4 cytology categories associated with DTS, as reported here, are similar to those obtained for rates associated with SurePath LBC on either the BD Totalys SlidePrep or the BD PrepStain Slide Processor (Supplementary Table 5).

The high level of acceptable cytology features reported in this study can be attributed to several factors. The use of SurePath LBC as a cell preservative is a feature of this process and the fact that DTS also uses a SurePath collected specimen results in a well-preserved sample for evaluation. Likewise, acceptable stain quality is to be expected, as the studies reported here all used standard SurePath LBC staining reagents from the manufacturer, thereby ensuring a high level of stain quality. As noted, the DTS process takes advantage of both features without any decrease in performance. Finally, both the DTS and SurePath LBC methods use comparable amounts of cellular material per slide. In the standard SurePath LBC process, 8 mL of the collected 10-mL specimen is processed over the density reagent, the enriched cell pellet is resuspended in 1 mL of water, and then 0.2 mL of the cell resuspension is applied to the slide. This represents approximately 16% of the collected cells deposited onto the slide through the settling chamber. Similarly, the DTS process deposits 2 mL of the collected SurePath specimen onto 1 mL of the density reagent, and following centrifugation, approximately 20% of the collected cells are deposited on the slide. Thus, both methods result in the application of comparable numbers of collected cervical cells on the slide.

### Limitations

The current study was a feasibility evaluation of the DTS method, designed to demonstrate acceptance rates for cervical cytology criteria following DTS slide preparation. The study was not designed to demonstrate clinical equivalence between DTS preparation and the standard SurePath LBC preparation methods. To show clinical equivalence, a split-sample study would be required to directly measure cellularity, impact of interfering substances (eg, blood or mucus), ratios of atypical squamous cells of undetermined significance (ASCUS) to LSIL, and concordance of detection rates for ASCUS, LSIL, ASC-H, and HSIL as outcomes between DTS and SurePath LBC preparation methods. Furthermore, the utility of DTS in routine cervical cancer screening and the detection of cervical precancer cells would need to be demonstrated using a split-sample study with SurePath LBC specimens collected within the intended cervical cancer screening population. Ideally, such a study would include the use of routinely collected cervical specimens from patients participating in a cervical cancer screening program, with comparison of matched SurePath LBC and DTS slides conducted for adequacy, unsatisfactory rates (ie, specific issues encountered with excessive blood, mucus, or inflammatory cells), and diagnostic equivalence. Clinical end points should include either adjudicated cytology for the SurePath LBC and DTS slides and/or biopsy results and the use of histopathology confirmed cervical intraepithelial neoplasia, grade 2 or worse (≥CIN2) lesions. The current study provides the data for feasibility, and the results justify a further comprehensive clinical evaluation.

## CONCLUSIONS

There have been several approaches to the development and validation of low-cost alternatives for cervical cytology screening.^13,22-24^ Although promising in initial studies, these low-cost systems do not appear to have progressed beyond the technical feasibility phase. As such, wider adoption of a technically and clinically validated low-cost system is still needed. These data demonstrate that high-quality LBC slides can be produced from this manual DTS method using only modified holders and a common tabletop centrifuge. This method retains the advantages of the SurePath LBC test, namely, standardized preparation, consistent morphology presentation, acceptable retention of critical cytology features, and detection of abnormal cells, with concordant cytology classification in comparison to the standard SurePath LBC preparation. Following additional clinical validation, laboratories with limited resources may benefit from incorporation of this manual LBC method.

## Supplementary Material

aqae068_suppl_Supplementary_Figures_S1-S4_Tables_S1-S5

## Notes

Funding: This work was sponsored by Becton, Dickinson and Company.

Conflict of interest disclosure: During the time of this study, Douglas P. Malinowski, Ryan Callaghan, Clark Whitehead, Romana Nelson, Lisa Allen, Dondrea Purnell, Adriann Taylor, Didier Morel, Aisha Dhewar, and Jeffrey Andrews were employed by the study sponsor, Becton, Dickinson and Company; BD Life Sciences—Integrated Diagnostic Solutions. Vanessa Soh Chai-Hong, Goh Giap-Hean, Soo-Yong Tan, and Sagar Tapas have nothing to disclose.
