# Supplementary material for: BD SurePath Direct to Slide (DTS) cervical cytology: Migrating the benefits of liquid-based cytology to low-resource settings
Source: Am J Clin Pathol. 2024 Jun 24;162(6):559–69. doi: 10.1093/ajcp/aqae068 (PMC11637525; doi:10.1093/ajcp/aqae068)
Supplement: aqae068_suppl_Supplementary_Figures_S1-S4_Tables_S1-S5 [file aqae068_suppl_supplementary_figures_s1-s4_tables_s1-s5.docx]

AJCP – 2024-01-0039 R1 – Malinowski – Revised Supplemental File – 3-22-2024

# SUPPLEMENTAL MATERIAL

## **TABLE S1**

| **Table S1**. Overall acceptance rate of DTS slides meeting the criteria for cellular features | | | |
| --- | --- | --- | --- |
|  | **Feasibility Study (**N = 96) | | |
| Cellular Feature | Number of acceptable slides | Percentage acceptable | 95% CI |
| Cellularity | 96 | 100% | (96, 100) |
| Cell distribution | 95 | 99% | (94, 100) |
| Cellular Preservation | 96 | 100% | (96, 100) |
| Stain Quality | 96 | 100% | (96, 100) |
| **Abbreviations:** DTS, direct to slide | | | |

## **TABLE S2**

| **Table S2.** Cellularity ranges of DTS preparation method in validation and reproducibility studies | | | | | |
| --- | --- | --- | --- | --- | --- |
| Cellularity Range | Site 1  Feasibility Study  (% of 96 Cases) | Site 1  Validation Study  (% of 384 Cases) | Site 2  Evaluation Study  (% of 300 Cases) | Average Percentage  (95% CI) |  |
| >90K | 1 | 0 | 5.3 | 2.10 (1.96, 2.24) |  |
| >40-90K | 31.2 | 21.6 | 71.7 | 41.50 (40.18, 42.82) |  |
| >5-40K | 67.7 | 78.1 | 22.3 | 56.03 (54.56, 57.51) |  |
| ≤5K | 0 | 0.3 | 0.7 | 0.33 (0.32, 0.35) |  |
| Total | 100% |  |  |  |  |
| Abbreviations: DTS, direct-to-slide | | | | | |

## **TABLE S3**

| **Table S3.** Concordance of Acceptable Cytology Features between DTS and SurePath LBC Preparations in the Split-Sample Study | | | |
| --- | --- | --- | --- |
| **Cytology feature** | **No. of DTS/SurePath LBC**  **concordants** | **% Concordants** | **No. DTS/SurePath LBC**  **discordants** |
| **Cellularity** | 493 | 98.6 | 7 |
| **Cell Distribution** | 497 | 99.4 | 3 |
| **Cellular Preservation** | 500 | 100 | 0 |
| **Stain Quality** | 500 | 100 | 0 |
| **Abbreviations:** DTS, SurePath LBC direct-to-slid | | | |

## **TABLE S4**

| Table S4. Detection of Infectious Organisms in Split-Sample Comparison of SurePath LBC and DTS Preparation Methods. | | |
| --- | --- | --- |
|  | No. Cases with Microorganisms | |
| Microorganism | DTS Preparation | SurePath LBC |
| Candida spp. | 12 | 16 |
| Actinomyces sp. | 3 | 3 |
| Trichomonas vaginalis | 1 | 1 |

## **TABLE S5**

| Table S5. DTS Feature Summary vs Totalys SlidePrep Product Insert* | | | | | | | | | | |
| --- | --- | --- | --- | --- | --- | --- | --- | --- | --- | --- |
| Slide Quality Feature | DTS Acceptance Rate  % [95% CI] (n/N) | |  | DTS Acceptance Rate: NILM Pool**^a^**  % [95% CI] (n/N) | | DTS Acceptance Rate: LSIL Pool**^a^**  % [95% CI] (n/N) | | DTS Acceptance Rate: HSIL Pool**^a^**  % [95% CI] (n/N) | | |
|  | Feasibility Study  (N=96) | Validation Study  (N=384) |  | BD SlidePrep  (N=120) | BD PrepStain  (N=120) | BD SlidePrep  (N=120) | BD PrepStain  (N=120) | BD SlidePrep  (N=120) | BD PrepStain  (N=120) |  |
| Cellularity | 100%  (96%, 100%) | 99.7%  (98.5%, 100%) |  | 100.0%  (96.9%, 100%) | 100.0%  (96.9%, 100%) | 100.0%  (96.9%, 100%) | 100.0%  (96.9%, 100%) | 100.0%  (96.9%, 100%) | 97.5%  (92.9%, 99.1%) |  |
| Cell distribution | 99% (95/96)  (94%, 100%) | 100% (99.0%, 100%) (384/384) |  | 100.0% (120/120)  (96.9%, 100%) | 100.0% (120/120)  (96.9%, 100%) | 100.0% (120/120)  (96.9%, 100%) | 100.0% (120/120)  (96.9%, 100%) | 100.0% (120/120)  (96.9%, 100%) | 100.0% (120/120)  (96.9%, 100%) |  |
| Cellular Preservation | 100% (96/96)  (96%, 100%) | 100% (99.0%, 100%) (384/384) |  | 100.0% (120/120)  (96.9%, 100%) | 100.0% (120/120)  (96.9%, 100%) | 100.0% (120/120)  (96.9%, 100%) | 100.0% (120/120)  (96.9%, 100%) | 100.0% (120/120)  (96.9%, 100%) | 100.0% (120/120)  (96.9%, 100%) |  |
| Stain Quality | 100% (96/96)  (96%, 100%) | 100% (99.0%, 100%) (384/384) |  | 100.0% (120/120)  (96.9%, 100%) | 100.0% (120/120)  (96.9%, 100%) | 100.0% (120/120)  (96.9%, 100%) | 100.0% (120/120)  (96.9%, 100%) | 100.0% (120/120)  (96.9%, 100%) | 100.0% (120/120)  (96.9%, 100%) |  |
| **Abbreviations:** | | | | | | | | | | |
|  | | | | | | | | | | |
| **^a^**Data reproduced from BD Totalys SlidePrep Product Insert, Table 14 ((reproducibility – Comparison of Acceptance rates with 95% CI between BD Totalys SlidePrep and BD PrepStain by Slide Quality Freature. | | | | | | | | | | |

## **FIGURE S1**

**Figure S1**


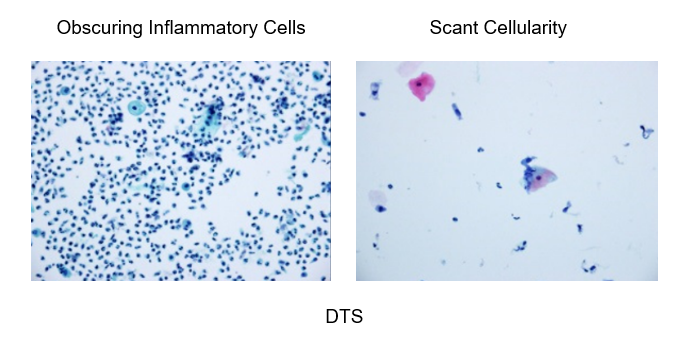


**Figure S1.** Images of Obscuring Inflammatory Cells and Scant Cellularity in DTS Preparations. The images of obscuring inflammatory cells is shown at 10X magnification and scant cellularity is shown at 20X magnification.

## **FIGURE S2**


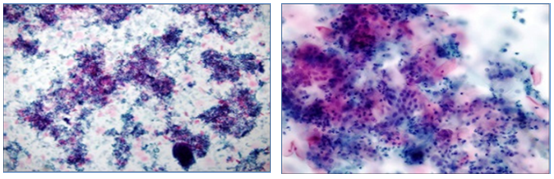


**Figure S2.** Images of Unacceptable Cell distribution Results in DTS Preparations. The images are shown at 4X magnification (left panel) and 20X magnification (right panel).

## **FIGURE S3**

**Figure S3**


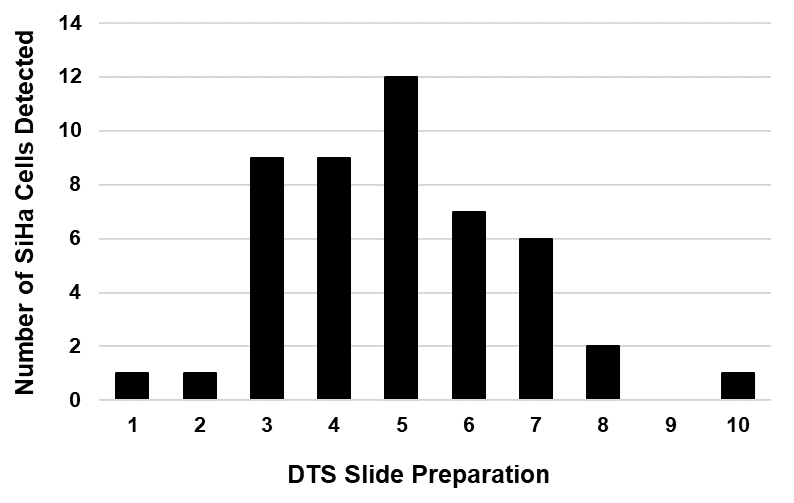


**Figure S3.** Rare event SiHa cell detection using the DTC preparation method. The SiHa cells spiked into a NILM pool were processed using the DTS method and the slides examined for the detection of SiHa cells. A total of 46 slides were prepared from the SiHa pool using the DTS preparation method. The expected number of SiHa cells per slide was approximately 2 cells. The observed range of SiHa cells detected was shown to be between 1 SiHa cell and 12 SiHa cells per slide. The median number of SiHa cells detected per DTS slide was 5 cells per slide. There were no DTS slides with 0 SiHa cells.

## **FIGURE S4**


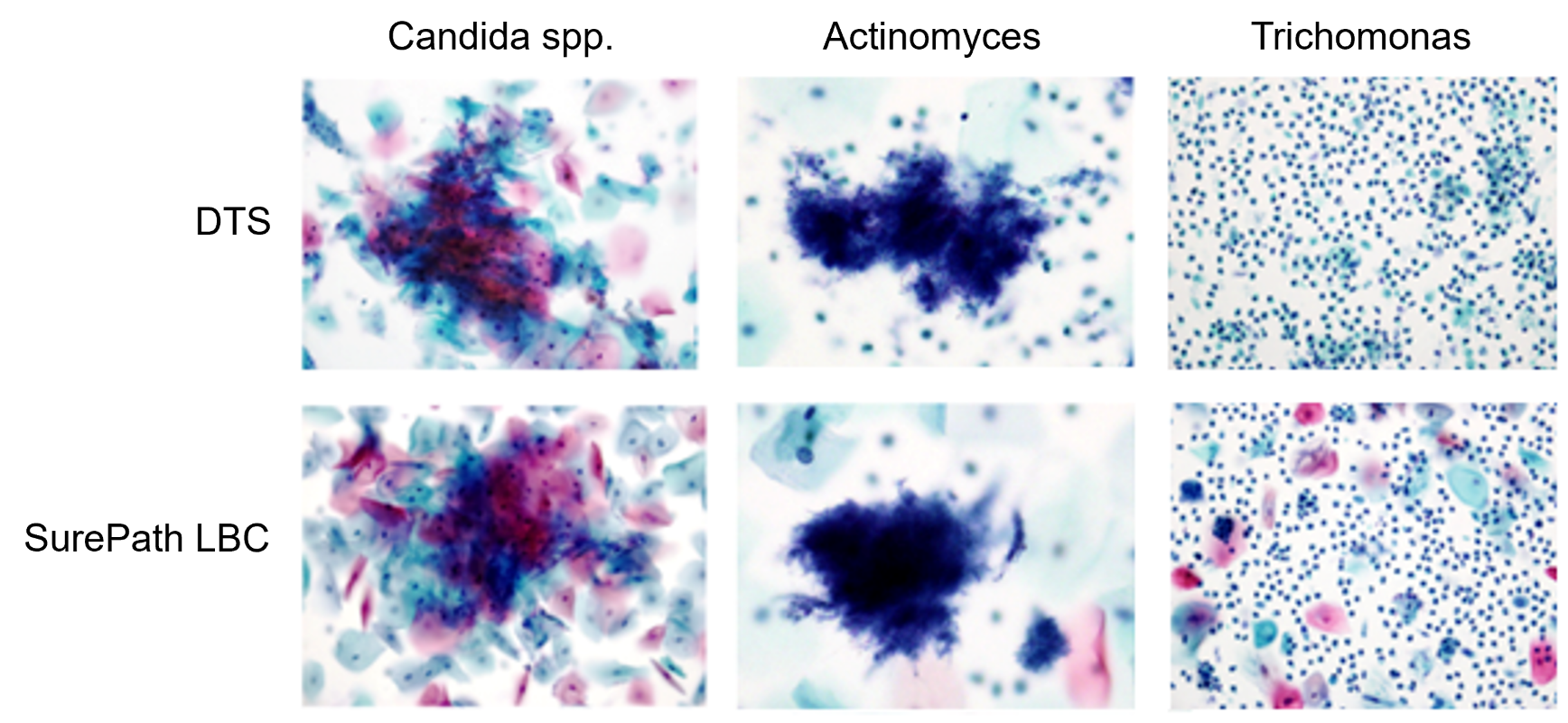


**Figure S4.** Microorganisms detected with DTS and SurePath LBC preparations in split-sample study. The images of Candida and Trichomonas are shown at 20X magnification. The images of Actinomyces are shown at 40X magnification.
